# Campbelltown – Changing our Future: study protocol for a whole of system approach to childhood obesity in South Western Sydney

**DOI:** 10.1186/s12889-019-7936-1

**Published:** 2019-12-18

**Authors:** Nicola Maitland, Mandy Williams, Bin Jalaludin, Steven Allender, Claudia Strugnell, Andrew Brown, Joshua Hayward, Nicholas Crooks, Jaimie Tredoux, Vincy Li, Karen Wardle

**Affiliations:** 1 Health Promotion Service, Population Health, South Western Sydney Local Health District, Liverpool, New South Wales Australia; 2 Population Health Intelligence, South Western Sydney Local Health District, Liverpool, New South Wales Australia; 3 Ingham Institute for Applied Medical Research, University of New South Wales, Sydney, New South Wales Australia; 4 Global Obesity Centre, Deakin University, Geelong, Victoria Australia; 5 Institute for Health Transformation, Deakin University, Geelong, Victoria Australia; 6 NSW Office of Preventive Health, Liverpool Hospital, Liverpool, New South Wales Australia

**Keywords:** Whole of system approach, Causal loop diagram, Community-based interventions, Community-led, Childhood obesity, Implementation science

## Abstract

**Background:**

In Australia, around 67% of adults and 25% of children (5–17 years) are currently overweight or obese (Australian Bureau of Statistics, 4364.0.55.001 - National Health Survey: First Results, 2017–18, 2018). The Campbelltown – Changing our Future study will translate ‘a whole of system’ approach, previously trialed in rural communities in Victoria and the Australian Capital Territoty, to Campbelltown Local Government Area (LGA), a socioeconomically and ethnically diverse urban community in south western Sydney, NSW.

**Methods:**

The study intervention will use a five-step approach; 1 – set up a childhood obesity monitoring system by collecting baseline data from children in primary schools across Campbelltown LGA to give a local context to the community when developing the systems map; 2 - key stakeholders develop systems maps which inform the development of the interventions; 3 - key stakeholders and community groups identify priority areas for action and form working groups; 4 - implementation of the interventions; 5 - evaluation of the interventions. The study will adopt a longitudinal pre/post design with repeated measures at baseline, 2 years and 4 years. Both qualitative and quantitative methods will be used to collect and analyse the data.

**Discussion:**

Addressing childhood overweight and obesity is complex and requires a multifaceted intervention. This approach has the capacity to impact a range of factors that influence childhood overweight and obesity utilising existing capacity of multiple partners with broad community reach. Findings will develop local responses which capture the complexity of obesity at a community level and further our understanding of the interrelationships and relative importance of local factors impacting childhood overweight and obesity. This study aims to provide evidence for systems methods and approaches suitable for adaption and scaling and may provide evidence of successful community intervention elements.

## Background

Tackling the rapid rise in overweight and obesity within Australia is a major challenge for the health system. In Australia, around 67% of adults and 25% of children (5–17 years of age) are currently overweight or obese [1]. Overweight and obesity are major determinants for many lifestyle related chronic diseases including type 2 diabetes, metabolic syndrome, cardiovascular disease and cancer [2]. Disadvantaged, Indigenous and some Culturally and Linguistically Diverse (CALD) populations are at greater risk [3]; for example, 33% of boys and 38% of girls from a low socioeconomic (SES) background are overweight or obese, compared to 22% of boys and 24% of girls from a high socioeconomic background [4]. The rise in obesity has resulted in increased rates of morbidity and mortality [5], costing the Australian health system more than $21 billion annually [6].

The World Health Organization (WHO) identified reducing obesity and diabetes as a voluntary global target in the Global Action Plan for the Prevention and Control of Non-Communicable Diseases 2013–2020 [7]. This recognition prioritises action towards addressing diabetes and obesity on a global scale [8]. Overweight and obesity in children impacts on both physical and mental health [9], particularly depressive symptomology [10], which has been shown to persist into adulthood [10]. Children who are overweight or obese are more likely to be obese adults and affected by obesity related comorbidities such as type 2 diabetes and hypertension [9].

The estimated prevalence of overweight and obesity among New South Wales (NSW) children is around 24%, and the rates in South Western Sydney Local Health District (SWSLHD) are higher (28%) [11, 12]. SWSLHD has been identified as the regional focus area for the 2015 NSW Premier’s Priority of reducing childhood overweight and obesity [12]. The Campbelltown – Changing our Future study is being conducted in Campbelltown Local Government Area (LGA), a large socioeconomically and ethnically diverse urban community within SWSLHD [13], characterised by higher levels of socioeconomic disadvantage. Campbelltown LGA spans across 31,000 ha of land with a current population of 168,000 people, the forecasted population growth is expected to reach over 275,700 people by 2036 [14].

A number of state-wide population level settings based initiatives to address healthy lifestyles in children are currently implemented across Campbelltown LGA. These include Munch and Move, Live Life Well @ School and Finish with the Right Stuff, which influence food and physical activity (PA) environments in the childcare, school and sport settings. These initiatives have two major drawbacks in light of the evidence. They recognise the influence of obesogenic environments, but they focus on single settings rather than taking a comprehensive approach to Campbelltown’s interrelated environments. They also lack community-based intervention components. Community-based interventions have been found to be both cost effective and an acceptable approach to government, community and industry [15].

The most recent Cochrane review for preventing obesity in children (aged 6–12 years) identifies that community-led, multi-component interventions that operate strategically across multiple levels of society show promise for obesity prevention [16]. Multi-component interventions are complex, offering a variety of factors to address a complex need or issue [17]. There are renewed calls for a ‘whole of system’ community-based approach to addressing the complex societal issue of obesity [18]. A systems based whole of community approach provides ways for communities to engage with multiple factors that influence societal pressures, the built environment, social capital and individual behaviours to identify opportunities for multi-faceted intervention within a community [2, 17, 19]. One of the key challenges to multi-component interventions is sustainability [20]. Community engagement is a key component of sustainability [20]. A whole of systems approach enables community engagement for community-based solutions to problems [21]. This approach can mobilise multiple sectors and organisations to enable multi-faceted change [22, 23]. For example, using the community-based system dynamics facilitation technique of group model building (GMB) invites stakeholders from multiple organisations, such as education, sport, health, local council and child care services to identify the local factors that influence childhood overweight and obesity. This approach accounts for complexity through considering the non-linear relationships between influential factors, feedback loops and unforeseen changes to the community setting (e.g. change of local government influencing environmental infrastructure) [24]. Involving stakeholders as part of this process in developing the initial causal loop diagram (CLD) creates a shared understanding.

The strength of this model lies in the community and stakeholder empowerment, allowing multi-faceted solutions to be designed using cross-sectoral collaboration under continually changing circumstances (e.g. changing Premier’s priorities and allocated funding), promoting opportunity for sustainable change [22, 23]. The Campbelltown – Changing our Future study aims to translate and evaluate a community-based systems approach to childhood overweight and obesity prevention, previously trialed in two Australian states/jurisdictions, Victoria and the Australian Capital Territory [2, 25], to Campbelltown LGA. The innovation of this work in the setting of Campbelltown – Changing our Future lies in the translation of the approach from a regional to an urban setting in addressing childhood obesity.

This paper will firstly discuss our protocol for how the ‘whole of system’ approach guides development and implements locally identified strategies which acknowledge the complexity of obesity. Secondly, this paper will outline our evaluation strategies to capture changes to children’s health, obesogenic environments and the resulting impacts of utilising a ‘whole of system’ approach.

We believe the Campbelltown – Changing our Future study will make a significant contribution to implementation science knowledge by contributing to the paucity of research studies that have used whole of community systems approaches and may provide evidence of successful community intervention elements.

## Methods/design

### Aim

The study aims to evaluate the effectiveness of a 'whole of system' approach to community based obesity prevention in Campbelltown LGA, a large socioeconomically and ethnically diverse urban community in south west Sydney, and evaluate its effectiveness in improving the local food and PA environments and behaviours. The main target audience will be primary school-aged children and their families living in Campbelltown LGA.

The research will address the following questions regarding childhood overweight and obesity prevention in the Campbelltown LGA:

1. How effective is a whole of system community-based approach in changing children’s dietary and PA habits?
2. To what extent does the whole of system community-based approach result in changes to local food and PA environments?
3. How effective is the whole of system community-based approach in decreasing body mass index (BMI) and the prevalence of childhood overweight and obesity?

### Study design

The study will adopt a longitudinal pre/post design with repeated measures at baseline, 2 years and 4 years, implemented over 5 years by the SWSLHD Health Promotion Service. Both qualitative and quantitative methods will be used to collect and analyse the data.

The study intervention will use a five-step approach; 1 – set up a childhood obesity monitoring system by collecting baseline data from children in primary schools across Campbelltown LGA to give a local context to the community when developing the systems map; 2 - key stakeholders develop systems maps which inform the development of the interventions; 3 - key stakeholders and community groups identify priority areas for action and form working groups; 4 - implementation of the interventions; 5 - evaluation of the interventions. The primary outcome measure will be BMI z-score and obesity prevalence in the community. Secondary outcome measures will include dietary information (fruit and vegetable intake, sugar sweetened beverage and water consumption) and PA behaviours (amount of PA and time spent on recreational screen time activities). Additional measures will include changes in community-based systems, policy and practice, the community’s capacity to identify, mobilise and address public health problems, and in built environments.

### Objectives and outcomes

The primary and secondary objectives and outcomes of the Campbelltown – Changing our Future study are outlined in Table 1. Each objective is to be measured and evaluated. Additionally, systems and environmental objectives will be measured to complement the outcomes seen with evaluation of the primary and secondary objectives. These objectives are tailored to inform the three research questions.

**Table 1:** Primary and secondary objectives and outcome measures

Objectives	Item to be measured	Proposed instrument/measure
Primary		
To examine change in • BMI-z score • childhood overweight and obesity prevalence	Anthropometry	Parent-reported height and weight
Secondary		
To examine changes in • sugar sweetened beverage intake • water intake • daily fruit and vegetable consumption • proportion of participants meeting the Australian Dietary Guidelines for fruit and vegetable intake	Diet	Modified version of the Simple Dietary Questionnaire (SDQ) [26]
• minutes per day spent in moderate to vigorous PA (MVPA) and Sedentary Behaviour (SB) • proportion of participants meeting national PA and screen time guidelines • mode of transport to and from school	PA and sedentary behaviour	Modified questionnaire containing items from Core Indicators and Measures of Youth Health [27] and SHAPES survey [28] Accelerometry data [29, 30]
• psychosocial health summary score • physical health summary score	Physical and psychosocial health	Paediatric Quality of Life Inventory (PedsQL) [31]
Systems and environmental		
To examine change in • school policy environment • school physical environment	Physical and social school environments	Live Life Well @ School desirable practices [32]
• built environment	Physical and social built environment	Community Park Audit Tool [33]
• communities’ understanding and preparedness to act on specific issues	Community capacity	Readiness to Change questionnaire [34]

### Data collection

The ‘whole of system’ approach requires the monitoring and evaluation of the intervention to be flexible. This paper will discuss the two main aspects for evaluating the effectiveness of the Campbelltown – Changing our Future study; 1 – children level changes; 2 – systems and environmental changes.

### Children level data collection

#### Population

The study population will be children aged 5–12 years attending primary school in Campbelltown LGA. The LGA has 42 primary schools (32 Government, 5 Catholic and 5 independently governed).

All children in Years 2 (7–8 years old), 4 (9–10 years old), and 6 (11–12 years old), at participating schools on the day of data collection will be eligible to participate.

#### Sample size

Sample size calculations are based on detecting differences in mean BMI z-score at the end of the study. Based on school enrolment data, we estimate there are 7560 children in years 2, 4 and 6 (based on an average of 180 eligible children per school) across the 42 primary schools in Campbelltown LGA. Assuming an 85% response rate using an opt-in approach process and that 70% of schools will participate, we expect to enrol more than 4400 children at each of the three waves of the study. From systematic reviews and previous studies that have evaluated the effect of whole of community interventions on children’s BMI z-scores [35, 36] we estimate BMI z-score standard deviation to be 0.93, the intra-cluster (school) correlation 0.04, and the average number of children measured per school 153. The target sample size (23 schools and 153 children per school) has a power of 80% of detecting a 0.11 difference of BMI z-score between groups (α = 0.05, two-sided test). This compares favourably with the observed z-score difference achieved in previous successful intervention studies in children of 0.16 [11]. At a population level, such reductions in community adiposity are meaningful and are likely to yield important health benefits for communities [16, 37].

#### Recruitment

An opt-in approach will be used for parents of students in Years 2, 4 and 6 to obtain parent-reported BMI. An opt-out approach will be used for students in Years 4 and 6 to complete the behavioural questionnaire and accelerometry.

Prior to baseline data collection, presentations will be made to school principal networks, followed by an information pack sent to each school principal, including a letter of invitation, study overview, design and protocol, and copies of the information statements, consent forms and student/parent surveys. It will explain that the study is aiming to evaluate long-term changes in weight and associated behaviours among school children in years 2, 4 and 6 in their community. The project manager will make an initial visit to each school to further clarify the study procedures with staff and to make assembly and classroom presentations of the study to students while distributing plain language statements and consent forms.

#### Protocol and procedures

Students in years 4 and 6 who have not returned an opt-out form will be invited to participate in the school-based data collection. Students will be asked to complete a 30 min questionnaire on electronic tablets during class time. The questionnaire will ask about their diet, PA and sedentary behaviour, and their physical and psychosocial health. On testing days, each participating child will be asked for verbal assent and should a child wish to withdraw at any time during data collection, they may do so without any consequence. Following survey completion, a sub-sample of students will be asked to wear a wrist-worn accelerometer for 7 days on their non-dominant hand. Students will be provided with basic care instructions and will be asked to wear the device at all times except for showering/bathing and combat sports, such as boxing, to reduce risk of injury/damage. At the conclusion of the 7 day period, students will be required to return the device to school administration staff.

The parent/carers of all students in years 2, 4 and 6 will be asked to complete the parent/carer questionnaire. Parents/carers will complete the questionnaire on their choice of paper form or electronically via an online survey tool. Completion of the survey will be considered implied consent. The questionnaire instrument will ask about basic child and family demographics, knowledge of national health recommendations and their children’s health habits. The questionnaire has been developed to complement the information captured in the student questionnaire with the inclusion of self-reported anthropometric measurements of their child/ren in years 2, 4 and 6. The model of parental proxy for younger students was adapted from the NSW SPANS survey [38].

Questionnaires will typically be conducted on a single day during a class period at each school in Term 1, 2019 (baseline), Term 1, 2021 (2 year follow up) and Term 1, 2023 (4 year follow up).

Ethical approval for this study was sought and granted from the SWSLHD Human Research Ethics Committee (HREC/17/LPOOL/314) and the State Education Research Applications Process (SERAP 2018274). Ethical approval was not granted for Catholic schools, therefore they were excluded from the sample.

#### Primary outcome measures and data management

##### Anthropometry: height and weight measurement

The current 2013 National Health and Medical Research Council’s (NHMRC) Clinical Practice Guidelines for the management of overweight and obesity in adults, adolescents and children in Australia recommend the use of BMI to classify overweight or obesity in Australian children and adolescents aged 2 to 18 years [39]. BMI is a weight-for-height index that is calculated by dividing weight (kg) by the square of height (m^2^) (kg/m^2^) [40]. In growing children and adolescents, BMI classification requires the use of age and sex-specific growth references [41]. To allow for international comparison, this study will use the WHO BMI growth reference which is an age-sex specific BMI z-score to classify overweight (> + 1 standard deviations) and obesity (> + 2 standard deviations) in young people aged 5 to 19 years [42].

Anthropometric measurements (height and weight) of children in years 2, 4 and 6 will be self-reported through the parent/carer questionnaire. A parent/carer instruction sheet and a paper measuring tape will be sent home with the questionnaire to aid parents/carers with accurate measuring of their child/ren’s height and weight [43]. Information will be included on the instruction sheet to assist parents in accessing their local General Practitioner or pharmacist in the case where they do not have access to a weight scale at home.

The effect of the intervention on the BMI z-score will be assessed using a linear mixed model with cluster as a random effect (school class) and time, intervention and interaction of time*intervention as fixed effects. Statistical analysis will be conducted on an intention-to-treat basis, assuming that all children in Campbelltown LGA will be exposed to the intervention. Missing outcome data (which we anticipate will be sparse due to the selection criteria “child present at school on the day of data collection”) will be managed using an inverse probability weighting approach.

#### Secondary outcome measures and data management

##### Diet quality: questionnaire

The promotion of the intake of healthy foods and reducing the intake of unhealthy foods and sugar-sweetened beverages by children and adolescents has been identified as one of the key recommendations in the WHO’s commissioned report on ending childhood obesity [44]. Food frequency questionnaires (FFQ) are the most commonly used tools to measure dietary intake in population level epidemiological studies [45]. They are cost effective and place a reduced burden on questionnaire respondents [46]. FFQs have been considered as one of the most reliable and age appropriate assessment methods of diet in children aged 11 years and younger [47]. A modified version of the Simple Dietary Questionnaire (SDQ) [26], based on the former (2003) Australian Dietary Guidelines [48, 49], has been selected to measure diet quantity and quality [26]. For fruit and vegetable consumption students are asked on a 15-point Likert scale the number of serves they typically consume per day (e.g. ‘none’, ‘0.5 serves’, ‘1 serve’, ‘1.5 serves’ etc.). Dichotomous variables indicating meeting or not meeting the Australian Dietary recommendations [48] of ≥2 serves of fruit and ≥ 5 serves of vegetables/day for children aged 9–11 years and ≥ 5.5 serves for boys aged ≥12-years will be created. For take-away food, snacks, sugar-sweetened beverages and dairy consumption students are asked to rate their frequency of usual daily consumption on an 8-point Likert scale (e.g. ‘3 or more times a day’, ‘2 times per day’ etc.). Usual daily water consumption is asked on a 6 point Likert scale (e.g. ‘none’, ‘1-2 glasses a day’, ‘3-4 glasses a day’ etc.), and usual daily breakfast consumption is asked on a 6-point Likert scale (e.g. ‘every day’, ‘almost every day’, ‘2-4 times a week’ etc.). Dichotomous variables will be created to examine change over time.

##### PA and sedentary behaviour: questionnaire and accelerometry

PA is favourably associated with physical, psychological, social and cognitive health outcomes amongst school-aged children and youth aged 5–17 years [50]. At a population level, PA and sedentary behaviour information is often obtained through self-report questionnaires as they are cost effective, easy to administer [51] and can provide group level estimates of PA [52]. A modified questionnaire containing three items from the standardised Canadian Core Indicators and Measures of Youth Health – Physical Activity & Sedentary Behaviour Module questionnaire [27] and 15 items from the School Health Action, Planning and Evaluation System (SHAPES) questionnaire [28] will be used to measure PA and sedentary behaviour in children aged 7–12 years. The modified version was adapted by Strugnell et al., (2013) for administration and measurement in grades four (9–10 years old) and six (11–12 years old) in the Healthy Together Victoria project [8]. This questionnaire will allow a 7-day recall of the total duration spent in moderate to vigorous PA (MVPA) and sedentary behaviour (outside of school) to be calculated, and allow adherence to Australia’s PA and sedentary behaviour guidelines for children aged 5–12 years of ≥60mins/day MVPA and ≤ 2 h screen time for recreation outside of school [29] to be determined.

To supplement self-reported behaviour, accelerometers will be used in a sub-sample of students to objectively measure minutes spent in light intensity PA, MVPA and sedentary time [30]. Accelerometers will be worn for 7 days on the non-dominant wrist. Activity counts will be accumulated over a 30 Hz sample rate and analysed using a 5-s epoch. Non-wear time will be calculated using the Toriano criteria of 60mins-day of consecutive zero’s with 1–2 min of tolerance [53]. Data will be considered valid if wear time is ≥500 min/day over a minimum of 3 days. Metabolic equivalent units (METs) will be assigned to classify PA intensity as sedentary (≤1.5 METs), light (1.5–2.9 METs) and moderate-to-vigorous (≥3.0 METs) using accelerometer cut-points for Axis-1 counts using the previously validated scores for use with this age group [54].

##### Physical and psychosocial health: questionnaire

The assessment of both psychosocial health and physical health is essential to provide an accurate reflection of the overall health of the individual [55]. The Pediatric Quality of Life Inventory 4.0 (PedsQL) is a 23 item questionnaire assessing four domains of health related quality of life; physical, emotional, social health and school [31]. Participants respond on a 5-point Likert scale to rate how much of a problem each statement is (e.g. ‘never’, ‘almost never’, ‘sometimes’ etc.). Physical health is asked over eight items (e.g. ‘It is difficult for me to walk more than 100 metres’), emotional health is asked over five items (e.g. ‘I feel afraid or scared’), social health is asked over five items (e.g. ‘I have trouble getting along with other children’) and school functioning is asked over five items (e.g. ‘It is hard to pay attention in class’). The PedsQL has been validated and has high internal consistency (α = 0.90–0.91) among children aged 8 – 11 years [31]. The scores of the 23 items are reverse scored and transformed to a score out of 100 for physical and psychosocial domains, with a higher score indicating better health related quality of life [56].

### Systems and environmental changes

#### Study Population

This study will use GMB to engage key leaders across Campbelltown LGA who are decision makers, experts and community members to facilitate cross-sectoral input into identification of influential factors and intrinsically driven interventions [23, 24]. These key leaders will be identified through partners in the project and existing contacts in Campbelltown LGA. Three GMB workshops will facilitate the development of a CLD to reflect their understanding of the relationships between influential factors across the LGA [23].

These key leaders and members of the community will be invited to attend three GMB workshops to develop the systems map for Campbelltown LGA. Additionally, key staff from Campbelltown local council and SWSLHD will be identified and invited to participate in GMB training to embed ongoing capacity to facilitate a whole of system approach in Campbelltown LGA [24]. The resulting systems maps will be used to inform the interventions that will influence local environments. The whole of Campbelltown LGA will be invited to participate in this aspect of the project, with evaluation to occur through multiple systems outcome measures.

#### Systems outcome measures

##### School environment: Live Life Well @ School checklist

The Live Life Well @ School (LLWatS) program is an important component of the NSW Government’s response to the prevention of childhood obesity. It is a key program that was established in 2008 [32], contributing to the NSW Premier’s Priority to reduce overweight and obesity rates of children by 5% over 10 years [12]. LLWatS is a joint initiative between the NSW Ministry of Health and the NSW Department of Education, in consultation with Catholic Schools NSW and the Association of Independent Schools of NSW [57]. It supports a range of internal and external PA and nutrition programs and resources across NSW [32]. The LLWatS program works with schools’ to support the implementation of recommended canteen/food service guidelines and general healthy eating principles, school curriculum supporting healthy eating and PA for all stages, school policies supporting healthy eating and PA. LLWatS staff monitor schools’ progress against a set of desirable practices.

##### Community capacity: questionnaire

Community capacity in this context refers to a “community’s ability to identify, mobilise and address public health problems” and will be investigated using a modified Readiness to Change (RTC) questionnaire [34]. The RTC questionnaire is a widely used tool that was developed for use before, during and after prevention programs to examine a community’s understanding and preparedness to act on a specific issue [58, 59]. This questionnaire will be completed at baseline, 2 year and 4 year follow-up with a purposive sample of the key stakeholders involved, including representatives from local council, business, organisations, cultural leaders and sporting bodies.

Responses to the RTC questionnaire used to measure changes in community capacity will be scored using descriptive statements on anchored scales and awarded a final RTC score. The final score will correspond to one of the nine stages of community readiness. This measure in community readiness will be examined for change with time and plotted against changes in the children’s dietary and PA behaviours at 2 year follow up, and also with obesity prevalence at 4 year follow up.

##### Built environmental: audits of the structural environment

Environmental audits will be conducted in the community at baseline, 2 years and 4 years using a modified Community Park Audit Tool (CPAT) developed by Active Living Research [33]. There will be a process evaluation measure of changes in the food and PA environments which, when used together with the other systems measures, will provide a comprehensive picture of changes made in the community setting and an explanation of how the intervention has worked. Environmental audit data collected from the community will be analysed descriptively and used to explain the changes made to the community setting as a result of the intervention.

### Limitations

The ideal study design would allow for anthropometrical measurements to be collected directly from primary school students by trained data collectors, however the study was unsuccessful in receiving the required ethical approval. Parent-report anthropometrical measures will be used instead. No comparison sample will be obtained, the data collected will be compared with data from surveys routinely conducted in NSW, for example, the NSW Population Health Survey which collects data on similar measures and includes school children from across NSW [11].

By necessity, CLDs that emerge from workshops are dependent on the participants who attend. This will reflect a particularly biased view of the system. To address this limitation, we will build maps iteratively over time with various groups in order to gain further perspective on the drivers.

This project only uses qualitative CLDs, which have limitations relative to quantified simulation models because of limitations in understanding of how components of complex systems interact without the aid of technology. Future research could build on this work with simulation to further clarify and strengthen community understanding.

## Discussion

The Campbelltown – Changing our Future study will focus on the effectiveness of a community-based 'whole of system' approach to changing food and PA environments, as well as the dietary, PA behaviours and BMI in children aged 5–12 years living in Campbelltown.

The community-based 'whole of system' approach facilitates the generation of new knowledge regarding implementation of obesity interventions in an urban community setting with a diverse multicultural community. This will be achieved by mapping perceived key drivers of local obesogenic systems as identified by the community. It is expected that this process will enable development of interventions to address the key drivers identified by the community through forming and maintaining sub-system working groups. The reflexivity of the approach allows for new evidence and strategies across the multiple factors that influence societal pressures, the built environment, social capital and individual behaviours to be applied quickly, effectively and efficiently [19]. Previous work in Victoria has seen communities develop over 300 activities across the broader community as demonstrated on the GenR8 Change website where the key leaders are empowering and strengthening their community to make healthy choices for people in the region, especially children [60].

This study applies rigorous data collection protocols and evaluation tools to collect data from multiple sources. The large anticipated sample size will support analyses that may deepen the understanding of the multiple factors and interactions relating to childhood obesity across an urban, socioeconomically diverse and multicultural community. This information can be used to further inform community-led interventions to tailor the work of sub-system working groups to local issues.

This study has the potential to make substantial improvements in child and adolescent health, dietary and PA behaviours and BMI. This approach allows for the sociodemographic factors of an urban, multicultural community with high levels of socioeconomic disadvantage to be considered when testing the effect of a whole of community approach overlaying the current state-wide population level settings based initiatives. Additionally, community-based interventions have been found to be both cost effective and an acceptable approach to government, community and industry [15]. Results of the study will provide further evidence about the effectiveness of this promising approach, with intention to improve the health of NSW children across a whole community. This information could be used by educators, policy makers and researchers in future efforts to improve the health of NSW children. This study may provide evidence for systems methods and approaches suitable for adaptation and scaling, and may provide evidence of successful community intervention elements. If the intervention is shown to be effective, it will be considered for scaling up and implementation across NSW.

## Data Availability

Not applicable.

## Abbreviations

BMI: Body Mass Index
CALD: Culturally and Linguistically Diverse
CLD: Causal loop diagram
FFQ: Food Frequency Questionnaire
GMB: Group model building
HREC: Human Research Ethics Committee
IOTF: International Obesity Task Force
kg: Kilograms
LGA: Local Government Area
LLWatS: Live Life Well @ School
MET: Metabolic equivalent units
MVPA: Moderate to vigorous physical activity
NHMRC: National Health and Medical Research Council
NSW: New South Wales
PA: Physical activity
PedsQL: Paediatric Quality of Life Inventory
RTC: Readiness to Change
SD: Systems dynamics
SDQ: Simple Dietary Questionnaire
SERAP: State Education Research Applications Process
SHAPES: School Health Action, Planning and Evaluation System
SPANS: Schools Physical Activity Nutrition Survey
SWSLHD: South Western Sydney Local Health District
WHO: World Health Organization
